# Polarized holographic lithography system for high-uniformity microscale patterning with periodic tunability

**DOI:** 10.1038/s41378-021-00256-z

**Published:** 2021-04-15

**Authors:** Gaopeng Xue, Qihang Zhai, Haiou Lu, Qian Zhou, Kai Ni, Liyu Lin, Xiaohao Wang, Xinghui Li

**Affiliations:** 1 Tsinghua Shenzhen International Graduate School, Tsinghua University, Tsinghua Campus, the University Town, Shenzhen, 518055 China; 2 Tsinghua-Berkeley Shenzhen Institute, Tsinghua University, Tsinghua Campus, the University Town, Shenzhen, 518055 China

**Keywords:** Micro-optics, Nanoparticles

## Abstract

Periodic microscale array structures play an important role in diverse applications involving photonic crystals and diffraction gratings. A polarized holographic lithography system is proposed for patterning high-uniformity microscale two-dimensional crossed-grating structures with periodic tunability. Orthogonal two-axis Lloyd’s mirror interference and polarization modulation produce three sub-beams, enabling the formation of two-dimensional crossed-grating patterns with wavelength-comparable periods by a single exposure. The two-dimensional-pattern period can also be flexibly tuned by adjusting the interferometer spatial positioning. Polarization states of three sub-beams, defining the uniformity of the interference fringes, are modulated at their initial-polarization states based on a strict full polarization tracing model in a three-dimensional space. A polarization modulation model is established considering two conditions of eliminating the unexpected interference and providing the desired identical interference intensities. The proposed system is a promising approach for fabricating high-uniformity two-dimensional crossed gratings with a relatively large grating period range of 500–1500 nm. Moreover, our rapid and stable approach for patterning period-tunable two-dimensional-array microstructures with high uniformity could be applicable to other multibeam interference lithography techniques.

## Introduction

Highly uniform, periodic micro- and nanoscale array structures are utilized in diverse applications involving photonic crystals^1–3^, optical metamaterials^4,5^, photodetectors^6,7^, and diffraction gratings^8–11^. Various types of diffraction gratings, such as blazed, ladder, planar, and concave gratings, have already been developed for application in monochromators^12^, spectrometers^13^, optical waveguides^14^, optical encoders^8–11,15^, and other optical instruments. In particular, two-dimensional (2D) crossed gratings with equal pitches along two orthogonal directions (the X- and Y-directions) are commonly employed in planar/surface optical encoder systems for multiaxis displacement measurements; these gratings are key components that provide a measurement reference. The movements of the grating with respect to the encoder’s reading head cause phase shifts of the ±1-order diffracted beams from the 2D crossed grating; these are converted into interference intensity changes. Consequently, a relative displacement with a high-resolution comparable to that from laser interferometers can be obtained upon analyzing the interference signals^16^. Inevitably, the overall performance of the optical encoder strongly depends on the 2D crossed-grating structural parameters. Specifically, the 2D grating pitches along the X- and Y-directions should exhibit consistency to achieve uniform ±1-order 2D diffraction efficiencies for higher signal-to-noise ratios of the interference signals. Moreover, the grating pitch must be as small as possible to reduce the interference signal period and improve the encoder system measurement resolution^8,17^.

Three major techniques, namely, mechanical ruling^18,19^, projection lithography^20^, and laser interference lithography^21–25^, have been developed for fabricating grating structures. The third of these, laser interference lithography, also known as maskless lithography, is a powerful technique for rapidly fabricating high-uniformity gratings with high-pitch flexibility. The technique can cover a fabrication range from submicrons to microns and offers the benefit of realizing optical configurations with high cost efficiency^26–29^. In general, two optical configurations are used in laser interference lithography: amplitude-division-based Mach–Zehnder interference lithography^26^ and wavefront-division-based Lloyd’s mirror interference lithography^27–29^. For the amplitude-division single-exposure system, two double-beam subsystems afforded by a two-axis diffraction unit can pattern the 2D crossed grating with only one exposure. However, this amplitude-division configuration requires complicated supplementary optics to adjust the light phase, which makes the optical system vulnerable to environmental disturbances. By contrast, the wavefront-division configuration, capable of dividing a single beam into multiple sub-beams via Lloyd’s mirror interferometer, permits a simple, compact, and stable optical system. A two-axis Lloyd’s mirror interference unit, configured with a grating substrate holder and two mirrors, can be utilized to fabricate 2D crossed gratings with a single exposure; this approach can eliminate the problem of undesired background light observed in two-step exposures and realize symmetric groove structures along the X- and Y-directions.

In the currently available configurations of two-axis Lloyd’s mirror interferometers, the nonorthogonal configuration based on the single-exposure method can be used to fabricate high-uniformity 2D crossed-grating patterns; however, an undesirable inclined morphology is generated along the grating depth direction owing to the asymmetric incidence of the two interference beams^30,31^. By contrast, the orthogonal configuration of two-axis Lloyd’s mirror interferometers, a corner-cube-like structure, can deliver symmetric interference beams to the grating substrate for fabricating symmetric structures along with the depth direction^32–37^. In terms of the fabrication mechanism based on the interference of multiply divided sub-beams, the two reflected sub-beams (X-beam and Y-beam) from the two orthogonally configured Lloyd’s mirrors each interact with the directly incident sub-beam (sub-beam 1) to form the desired 2D crossed-grating patterns. However, it is necessary to eliminate the additional interference between the two reflected sub-beams to obtain high-uniformity structures. In our previous studies, we systematically analyzed the spatial polarization states of the two reflected sub-beams and determined the optimal exposure conditions to automatically eliminate the additional interference at certain incident angles. Subsequently, we realized a large-area 2D crossed grating (20 mm × 20 mm) with a ∼1 µm-level period without polarization modulation^34,35^. Note that to fabricate 2D crossed gratings with adjustable periods, the initial-polarization angles of the multiple sub-beams at their corresponding incident angles must be precisely modulated to effectively eliminate additional interference fringes. Previous studies have shown that multiply divided sub-beams with elliptical and linear polarization states can be modulated to realize high-uniformity 2D crossed gratings^36,37^; however, the exposure system achieves elliptical polarization modulation with two half- and quarter-wave-plate modulators, which complicates the optical path and constrains the overall area of the fabricated 2D crossed grating. On the other hand, the linear polarization modulation scheme with a simple optical system configured with only two half-wave-plate modulators is more attractive for practical application. Moreover, very few studies have systematically and comprehensively analyzed linear polarization modulation to realize interference fringes with adjustable periods.

In this study, we thoroughly analyze a laser interference lithography system based on an orthogonal two-axis Lloyd’s mirror configuration and systematically trace the evolution of the spatial polarization states of multiply divided sub-beams after interaction with the interference unit. A mechanism for optimizing the initial-polarization states of multiple sub-beams in the arbitrary incident angle range of [0°–90°] is established, mainly based on the nonorthogonality of the polarization states between two reflected sub-beams; this setup eliminates additional interference fringes and yields the preferred 2D crossed grating with the corresponding period. We use the degree of nonorthogonality between the two reflected sub-beams and identical interference intensity contrasts between two crossed directions as indices to study polarization modulation effects. Finally, we determine the optimal polarization angle combinations of the multiply divided sub-beams and validate our simulations with experimental results for different grating periods.

The remainder of this paper is organized as follows: Section 2 systematically establishes the polarization modulation theoretical model of the orthogonal two-axis Lloyd’s mirror interferometer to generate a 2D crossed grating with high uniformity. Section 3 describes the experimental setup of the exposure system used to verify the simulation results. Section 4 summarizes our findings and outlines our proposed future work.

## Results

### Polarization modulation theory

**Fig. 1:**
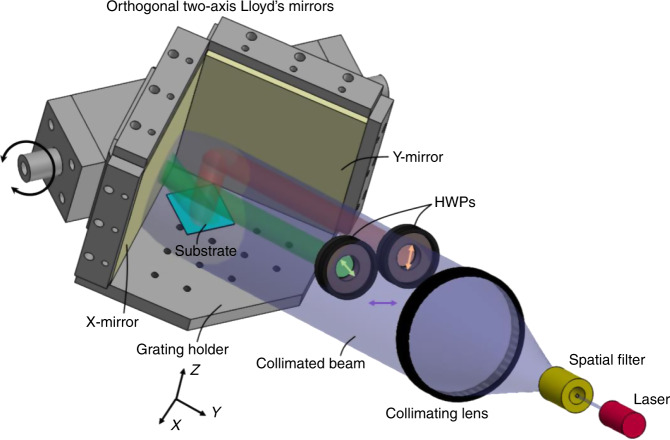
Optical configuration schematic of the interference exposure system based on the orthogonal two-axis Lloyd’s mirror interference unit. *HWP* half-wave plate.

Figure 1 shows the optical configuration of the interference exposure system, which is composed of a laser source, a beam-shaping module, half-wave plates (HWPs), and an orthogonal two-axis Lloyd’s mirror interference unit. The laser source provides a stably polarized initial incident beam of a suitable wavelength. The beam-shaping module consists of a spatial filter and a collimating lens to magnify the incident beam to a certain divergence angle, filter the edge portion of the divergent beam, and collimate the shape of the divergent beam from a spherical to a plane wave. The orthogonal two-axis Lloyd’s mirror module consists of two mirrors (X- and Y-mirrors) and a grating holder, which are positioned edge to edge and orthogonally to each other. Typically, the collimated beam, projected onto the interferometer unit via the wavefront-division mechanism, is divided into five sub-beams: one directly projected sub-beam, two sub-beams once-reflected by the X- and Y-mirrors, and two sub-beams twice-reflected successively by the X-/Y-mirrors and Y-/X-mirrors. It should be noted that the directly projected sub-beam interfering with the two once-reflected sub-beams can realize the desired 2D crossed-grating patterns. For this purpose, the two twice-reflected sub-beams must be blocked with physical filters to eliminate the extra generated stripes in the interference field^34–37^. The two HWPs, located between the collimating lens and the interference unit, are employed to adjust the polarization orientation angle of the two once-reflected sub-beams to determine the optimal initial-polarization-state combination of the three sub-beams of interest. The HWPs play an integral part in the optimization required to realize the preferred 2D interference fringes in this approach.

**Fig. 2:**
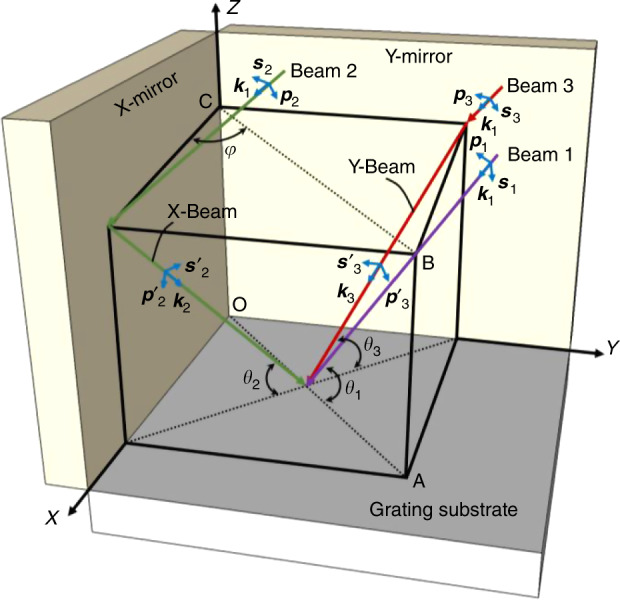
3D polarization tracing model of the three sub-beams of interest interacting with the orthogonal two-axis Lloyd’s mirror interference unit. Here, ***p*** denotes a unit vector in the incident plane perpendicular to wave vector ***k***, and ***s*** is a unit vector perpendicular to the incident plane.

As the polarization states of the sub-beams play a key role in generating the interference stripes, we need to systematically trace the evolution of the spatial polarization states of the sub-beams after their interaction with the two-axis Lloyd’s mirrors. Figure 2 shows the 3D polarization tracing model of the three sub-beams of interest interacting with the orthogonal two-axis Lloyd’s mirror interference unit in the context of their initial-polarization states and the polarization effects of the X- and Y-mirrors. Here, we construct a Cartesian coordinate system {X, Y, Z} as the global coordinates for the orthogonal two-axis Lloyd’s mirror interference unit. To describe the transformation relationship of the polarization states of the three sub-beams due to their interaction with the interferometer unit, a local coordinate system {***p***, ***s***, ***k***} for each sub-beam is commonly established, where ***p*** denotes a unit vector in the incident plane perpendicular to wave vector ***k*** and ***s*** denotes a unit vector perpendicular to the incident plane. In the 3D polarization tracing model^35,36^, the initial-polarization vectors of the three sub-beams first need to be standardized in the global coordinates; then, the standardized polarization vectors of the two once-reflected sub-beams must be further transformed into the corresponding local coordinates for calculation of the polarization effects of the two X- and Y-mirrors. Finally, the two vectors affected by the polarization effect are again transformed from local to global coordinates to recover the standardized polarization states. The direction vector of the three incident sub-beams is located in plane OABC with an angle *φ* = 45° with respect to the X-mirror. Parameter *θ* represents the incident beam angle with respect to the grating substrate.

When the projection of the three sub-beams (X-beam, Y-beam, and sub-beam 1) is superimposed on the grating, the total electric vector can be presented as

1 $${\boldsymbol{e}}({\boldsymbol{r}},t) = \mathop {\sum}\limits_{m = {\mathrm{1}}}^3 {A_m{\boldsymbol{E}}_m \cdot {\Re} \{ \exp [i(k{\boldsymbol{k}}_m \cdot {\boldsymbol{r}}) - \omega t + \delta _m]} \} ,$$

where *m* denotes a positive integer (=1, 2, 3), *A* is the amplitude of the electric field, ***E*** = [*E*_*x*_, *E*_*y*_, *E*_*z*_]^T^ (the 3D Jones vector^38^) is the unit vector of polarization, $\Re${·} is the real part of a complex number, and *i* is the imaginary unit. Moreover, *k* = 2π*n*/*λ* denotes the wavenumber (where *n* denotes the refractive index (=1 in air) and *λ* is the wavelength), ***k*** is the unit vector of the wave, ***r*** = [*x*, *y*, *z*]^T^ is the position vector in global coordinates, *t* is the time, *ω* is the angular frequency, and *δ* is the phase. Therefore, the total intensity distribution *I* of the 2D interference fringes, resulting from the three sub-beam superposition can be obtained as

2 $$I({\boldsymbol{r}}) = {\boldsymbol{e}}({\boldsymbol{r}},t) \cdot [{\boldsymbol{e}}({\boldsymbol{r}},t)]^ \ast = \mathop {\sum}\limits_{m = 1}^3 {A_m^2} + 2\mathop {\sum}\limits_{m = {\mathrm{1}}}^{\mathrm{2}} {\mathop {\sum}\limits_{m < n} {A_mA_n{\boldsymbol{E}}_m \cdot {\boldsymbol{E}}_n^ \ast \cos [k({\boldsymbol{k}}_m - {\boldsymbol{k}}_n) \cdot {\boldsymbol{r}} + (\delta _m - \delta _n)]} } ,$$

where *n* = 2 or 3. From Eq. (2), we can derive the periods of the 2D crossed grating along with two orthogonal directions as

3 $$g_{12} = g_{13} = \frac{{2\pi }}{{k\left| {{\boldsymbol{k}}_1 - {\boldsymbol{k}}_2} \right|}} = \frac{{2\pi }}{{k\left| {{\boldsymbol{k}}_1 - {\boldsymbol{k}}_{\mathrm{3}}} \right|}}{\mathrm{ = }}\frac{\lambda }{{\sqrt 2 \cos \theta }},$$

where *g*_12_ and *g*_13_ are the grating periods from the X- and Y-beams interfering with sub-beam 1, respectively. We note here that the generated interference fringes depend on wave vector ***k***_*m*_ and polarization vector ***E***_*m*_. These are in turn determined by direction angles *θ* and *φ* (45°), the initial-polarization states ***E***_0*m*_ of sub-beams 1–3, and the polarization effects of the two mirrors (which are characterized by the Jones matrix and depend on the properties of the coating layers on the mirrors^39^).

To fabricate a high-uniformity 2D crossed grating of interference stripes along the X- and Y-directions, two conditions need to be satisfied simultaneously: (a) the additional interference between the X- and Y-beams must be eliminated, as it can affect the desired interference stripes; (b) the interference fringes generated by the interaction of the X- and Y-beams with sub-beam 1 must be identical. Condition (a) implies that the polarization states of the X- and Y-beams reflected from the two mirrors should be orthogonal. Therefore, we introduce the nonorthogonality degree *D*_*mn*_ (*D*_12_, *D*_13_, and *D*_23_) to qualitatively analyze the interference intensity between any two beams. When the nonorthogonality of the polarization vectors between two beams satisfies

4 $$D_{mn} = \left| {{\boldsymbol{E}}_m{\boldsymbol{E}}_n^ \ast } \right| = \left| {{\boldsymbol{E}}_n{\boldsymbol{E}}_m^ \ast } \right| = 0,$$

the relationship of the polarization angles between the two beams is orthogonal. In the equation, the superscript * denotes the Hermitian conjugate. To obtain the optimal solution corresponding to condition (a), we adopt a traversal comparison method to estimate the minimal value of the nonorthogonality degree *D*_23_ in the full polarization angle range of [−90°–90°] at each incident angle in the range of [0°–90°]. In contrast, condition (b) implies that the interference fringe contrast *C*_12_ for the interference between the X-beam and sub-beam 1 should be identical to the interference fringe contrast *C*_13_ for the interference between the Y-beam and sub-beam 1, i.e.,

5 $$C_{12} = C_{13}.$$

According to Eq. (2), the interference intensity *I*_*mn*_ and the interference intensity contrast *C*_*mn*_ for the interference between any two beams can be obtained as

6 $$I_{mn} = A_m^2 + A_n^2 + 2A_mA_n{\boldsymbol{E}}_m \cdot {\boldsymbol{E}}_n^ \ast \cos [k({\boldsymbol{k}}_m - {\boldsymbol{k}}_n) \cdot {\boldsymbol{r}} + (\delta _m - \delta _n)],$$

7 $$C_{mn} = \frac{{I_{\max } - I_{\min }}}{{I_{\max } + I_{\min }}} = \frac{{2A_mA_n{\boldsymbol{E}}_m{\boldsymbol{E}}_n^ \ast }}{{A_m^2 + A_n^2}},$$

where *I*_max_ and *I*_min_ denote the maximum and minimum values of the interference fringe intensity, respectively. Owing to our wavefront-division mode, the amplitude values of the divided sub-beams are identical. Therefore, Eq. (7) can be further simplified to

8 $$C_{mn} = D_{mn},$$

meaning that the nonorthogonality degree *D*_*mn*_ can also determine the contrast of interference intensities.

To obtain the optimal combination of the initial-polarization states of sub-beams 1–3, we need to solve Eqs. (4) and (5), corresponding to conditions (a) and (b). First, the initial-polarization states of sub-beams 1–3 are simply assumed to be linear, corresponding to ***E***_0*m*_ = [cos*α*_*m*_, sin*α*_*m*_, 0]^T^, where *α*_*m*_ (*m* = 1, 2, 3) represents the polarization angle in the range of [−90–90°]. Here, the polarization angle of 90° with the corresponding polarization vector of [0, 1, 0]^T^ corresponds to *s*-polarization, equal to the output polarization angle of the laser source used in our experiments. Next, we adopt two routes to obtain the optimal solutions based on the implementation order of conditions (a) and (b). In route 1, we first implement condition (a) by estimating the minimal value of *D*_23_ (corresponding to the unique polarization angles *α*_2_ and *α*_3_) via the traversal comparison method at each incident angle. Subsequently, we implement condition (b) by obtaining the polarization angle *α*_1_ based on the minimal difference in the interferdenotes a positive integer ence intensity contrasts *C*_12_ and *C*_13_. Here, we set the minimal difference to be ≤10% to guarantee that the differences in the interference intensity contrasts are reasonably small. Finally, we choose the optimal polarization angle *α*_1_ for the maximum values of *C*_12_ and *C*_13_ corresponding to the maximum values of *D*_12_ and *D*_13_ to achieve high intensities of the desired interference fringes. By contrast, in route 2, sub-beam 1 is assumed to be in the same polarization state as the laser source, and only the polarization states of sub-beams 2 and 3 need to be modulated by two HWPs to satisfy the above requirements. Owing to the above precondition of *α*_1_ = 90°, we can determine that *α*_2_ = −*α*_3_ is a solution to *C*_12_ = *C*_13_, which completely fulfills condition (b). On this basis, we implement condition (a) by determining the optimal polarization angles *α*_2_ and *α*_3_ via the traversal comparison method for each incident angle. The polarization and incident angles in the following traversal comparison are both increased in steps of 0.1° to achieve more accurate results.

**Fig. 3:**
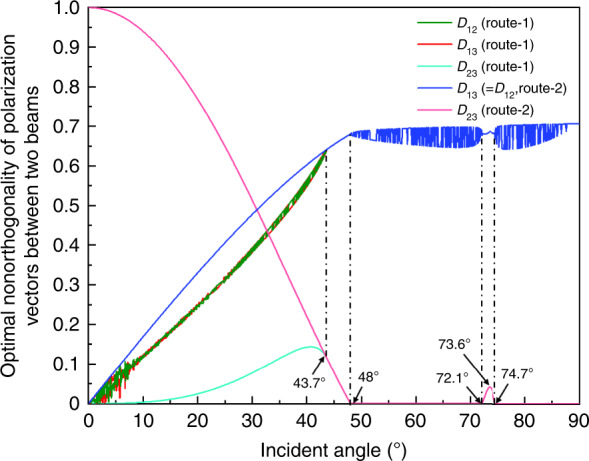
Optimal nonorthogonality of the polarization vectors of two beams at different incident angles based on the two routes of different implementation orders of two decisive conditions. Here, *D*_12_, *D*_13_, and *D*_23_ represent nonorthogonality values of the polarization vectors among subbeams 1–3.

Figure 3 shows the traversal comparison calculation results for the selected optimal nonorthogonality degrees (*D*_12_, *D*_13_, *D*_23_) of the polarization vectors of any two beams at different incident angles as per the implementation order of the two routes corresponding to conditions (a) and (b). In the incident angle range of [0°–43.8°], the nonorthogonality degree *D*_23_ of route 1 exhibits smaller values than that of route 2, which implies that route 1 more effectively eliminates the influence of the additional interference. Moreover, the nonorthogonality degrees *D*_12_ and *D*_13_ of route 1, increasing from 0 to 0.68, exhibit nonidentical values with a certain variation (<10%), which further leads to nonidentical interference intensity contrasts *C*_12_ and *C*_13_. Nevertheless, the selected optimal nonorthogonality degrees (*D*_12_, *D*_13_, *D*_23_) of the polarization vectors between routes 1 and 2 have the same values in the remaining incident angle range of [43.8°–90°]. Upon further narrowing the incident angle range to [48°–90°], we note that the system enters a stable regime that guarantees the realization (and hence fabricability) of the 2D interference fringes. This situation arises owing to the smallest nonorthogonality *D*_23_ (on the order of 10^−4^ or smaller) and a high nonorthogonality *D*_12_ (= *D*_13_) with a variation range from 0.64 to 0.71. In this incident angle regime, the realizable periods (referring to Eq. (3)) of the 2D crossed grating based on the laser source wavelength (*λ* = 442 nm) are >467 nm; these values can easily satisfy the conventional requirements of optical encoder systems with a grating period range from 0.5 to 1.5 μm. We note that there is a small raised “bump” corresponding to nonorthogonality *D*_23_ within the incident angle range of [72.1°–74.6°], where the peak (0.042) of nonorthogonality *D*_23_ corresponds to the incident angle of 73.6°.

**Fig. 4:**
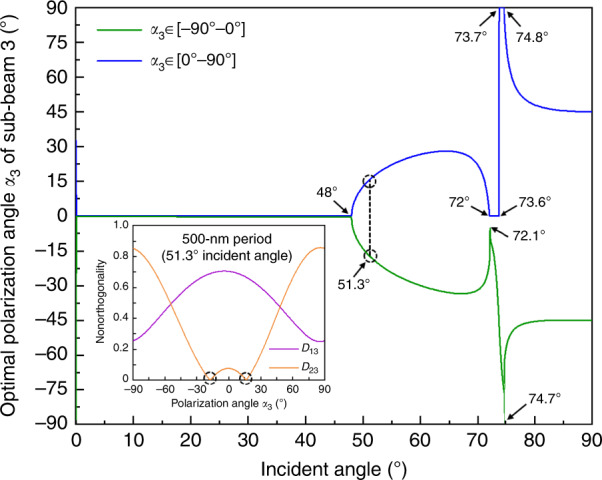
Optimal polarization angles of sub-beam three for different incident angles based on the route two implementation. Inset: an example of nonorthogonality values of the polarization vectors in the whole polarization angle range at an incident angle of 51.3°. Here, *α*_3_ denotes the polarization angle of sub-beam 3, and *D*_13_ and *D*_23_ represent nonorthogonality values of the polarization vectors among sub-beams 1–3.

To further examine the distribution trend of the optimal nonorthogonality of the polarization vectors, we plot the corresponding optimal polarization angles at each incident angle in Fig. 4. We adopt route 2 as our actual implementation scheme, as it requires a simpler optical system with only two HWPs to realize polarization modulation and identical interference intensity contrasts *C*_12_ and *C*_13_. For each incident angle in the entire range of [0°–90°], we select the optimal polarization angles *α*_3_ (=−*α*_2_) of sub-beam 3 with a fluctuation distribution via the traversal comparison method within the full polarization angle range of [−90°–90°]. For an incident angle of 51.3° (corresponding to a grating period of 500 nm), the inset shows two minimal values (2.99 × 10^−4^@−17.7° and 2.52 × 10^−4^@16.2°) of nonorthogonality *D*_23_ distributed in the negative and positive polarization angle ranges of [−90°–0°] and [0°–90°], respectively. Relative to these two minimal values, at each incident angle, it should be mentioned that the selected optimal polarization angles between two adjacent incident angles may distribute in different regimes in the ranges of [−90°–0°] and [0°–90°]. Among the fabricable regimes of the 2D crossed grating with an incident angle range of [48°–90°], there is a “mutation” region in the range of [72°–74.8°] where the derivative of the optimal polarization angle with respect to the incident angle contains discontinuities; this corresponds to the small shoulder region in Fig. 3. Furthermore, there exists a “special” range of [73.7°–74.8°], wherein the optimal polarization angle of sub-beam 3 is ~90°, i.e., *s*-polarization. This means that, in this range, the additional interference between the X- and Y-beams could be nearly eliminated without polarization modulation, as has already been demonstrated in our previous research work^35^.

### Exposure system experimental setup

**Fig. 5:**
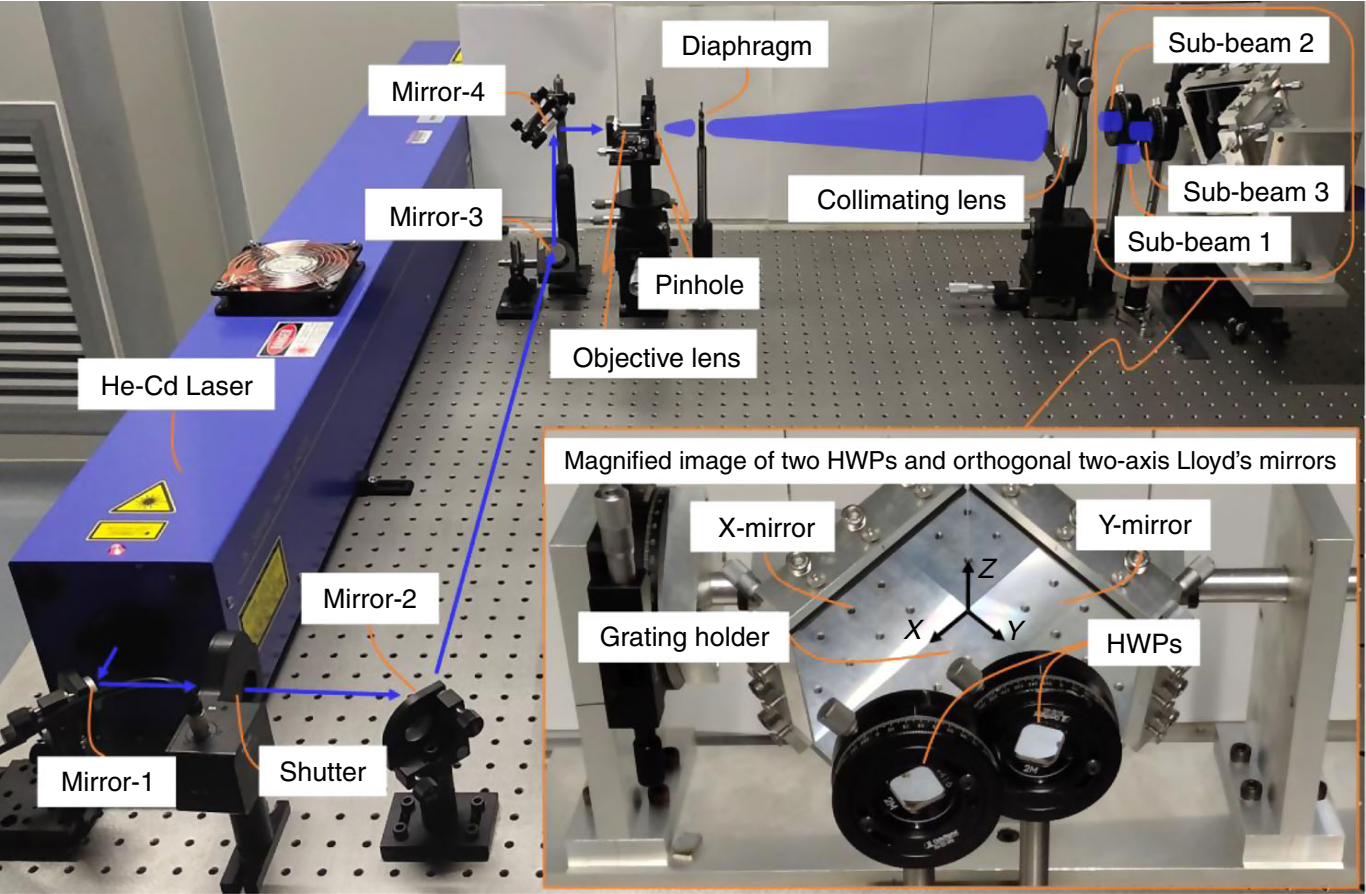
Experimental setup of the exposure system. Inset: magnified image showing the two half-wave plates and orthogonal two-axis Lloyd’s mirror interference unit. Here, the orthogonal two-axis Lloyd’s mirror module consists of two mirrors (X- and Y-mirrors) and a grating holder, and mirrors 1–4 act on guiding the output beam of the He-Cd laser. *HWP*: half-wave plate.

To experimentally demonstrate the feasibility of our polarization modulation model of the orthogonal two-axis Lloyd’s mirror interferometer, we setup an exposure system consisting of a laser source, a beam-expanding unit, two HWPs, and a two-axis Lloyd’s mirror interference unit (Fig. 5). Based on its stable polarization property and the photoresist (PR) sensitivity to the laser wavelength, we adopted a commercial He–Cd laser (KIMMON KOHA) with a 180-mW power and a 442-nm wavelength as our laser source. The output laser beam, continuously reflected by mirrors 1–4, enters the beam-expanding unit, which consists of an objective lens with a 3.3-mm working distance, a 10-μm-diameter pinhole, and a collimating lens (effective focal length = 500 mm). A diaphragm is utilized to cutoff the edge portion of the expanded circular beam. Subsequently, the resulting 75-mm-diameter collimated beam is divided via the two HWPs into sub-beams 1, 2, and 3, which are finally projected onto the grating, X-mirror, and Y-mirror, respectively. The initial-polarization states of sub-beams 2 and 3 can be manipulated by the two HWPs with an adjustment accuracy of 1°. These HWPs are placed with an overlapping edge to ensure a relatively large area of interference fringes. The grating holder and two mirrors, perpendicular to each other, compose the two-axis Lloyd’s mirror interference unit. The two aluminum (Al)-coated mirrors have a 95% reflectivity and a *λ*/10 surface flatness to cater to our wavelength range of interest. The orthogonal two-axis Lloyd’s mirror interference unit can be precisely rotated to an arbitrary incident angle to obtain interference with the required grating period.

### Fabrication results and evaluation

Table 1 presents simulated and experimental interference fringes of the 2D crossed gratings realized with different initial-polarization-state combinations of sub-beams 1–3 for different grating periods of 500, 750, 1000, 1250, and 1500 nm. Notably, larger incident angles to fabricate larger grating periods cause smaller interference areas of the three sub-beams in the orthogonal two-axis Lloyd’s mirror interferometer, especially larger than 78° (>1500 nm). To compare the polarization modulation effects, we applied three initial-polarization-state combinations of sub-beams 1–3 for each grating period: first, zero polarization modulation; second, minimal nonorthogonality of *D*_23_ with respect to polarization angle *α*_3_ (=-*α*_2_) of sub-beam 3 in the range of [−90°–0°]; and third, the same as before but in the range of [0°–90°]. A triplet of orientation angles (*α*_1_, *α*_2_, *α*_3_) was used to represent the initial-polarization-state combination of sub-beams 1–3, where (90°, 90°, 90°) indicates the special case without polarization modulation. In this special situation, the simulated interference fringes exhibit slightly irregular patterning, which can be attributed to the nonorthogonality value *D*_23_. For example, *D*_23_ varies between a maximum of 0.85 and a minimum of 0.06 between the periods of 500 and 1250 nm, thereby resulting in different degrees of the irregularity of the interference fringes. By contrast, the simulated interference fringes with optimal combinations of the initial-polarization angles in the regimes of [−90°–0°] and [0°–90°] produce relatively high-uniformity 2D crossed gratings. In this study, we used an atomic force microscope (Bruker Dimension Icon) in peak tapping mode to evaluate the characteristics of the fabricated 2D grating surface morphologies with a scanning area of 5 μm × 5 μm. We note here that the red and blue regions in the simulated images represent high and low interference intensities, respectively, and the bright regions in the experimental images indicate the remaining PR (owing to our use of positive PR). Despite possible fabrication errors related to the accuracy of the two HWPs, calibration effect of the zero incident angle of the interference unit, etc., the realized interference fringes of the 2D crossed gratings exhibit good agreement with the simulated results at different grating periods for different initial-polarization-state combinations of sub-beams 1–3. These results demonstrate that our systematic polarization manipulation method applied to the two-axis Lloyd’s mirror interference system is a promising approach to fabricating high-uniformity 2D crossed gratings over the relatively large grating period range of 500 to >1500 nm. We note that this range more than satisfies the requirements of commonly employed planar/surface optical encoder systems.

**Table 1:** Comparison of simulated and experimental interference fringes of 2D crossed gratings for different initial-polarization-state combinations of the three sub-beams and different grating periods.

Grating period (incident angle)	Simulated and experimental interference fringes of 2D crossed gratings		
	Nonorthogonality of *D*_23_ without polarization modulation	Minimal nonorthogonality *D*_23_ @ (*α*_1_, *α*_2_, *α*_3_) with *α*_3_ in the range of [−90°–0°]	Minimal nonorthogonality *D*_23_ @ (*α*_1_, *α*_2_, *α*_3_) with *α*_3_ in the range of [0°–90°]
500 nm (51.3°)			
	0.85 @ (90°, 90°, 90°)	2.99 × 10^−4^ @ (90°, 17.7°, −17.7°)	2.52 × 10^−4^ @ (90°, −16.2°, 16.2°)
750 nm (65.4°)			
	0.52 @ (90°, 90°, 90°)	3.23 × 10^−4^ @ (90°, 33.1°, −33.1°)	1.54 × 10^−4^ @ (90°, −28°, 28°)
1000 nm (71.8°)			
	0.2 @ (90°, 90°, 90°)	4.41 × 10^−5^ @ (90°, 23.3°, −23.3°)	4.62 × 10^−5^ @ (90°, −5.9°, 5.9°)
1250 nm (75.5°)			
	0.06 @ (90°, 90°, 90°)	8.25 × 10^−5^ @ (90°, 52.9°, −52.9°)	1.41 × 10^−4^ @ (90°, −66.1°, 66.1°)
1500 nm (78°)			
	0.26 @ (90°, 90°, 90°)	5.35 × 10^−5^ @ (90°, 46.7°, −46.7°)	7.89 × 10^−5^ @ (90°, −51.3°, 51.3°)

Finally, we systematically established a linear polarization modulation mechanism in orthogonal two-axis Lloyd’s mirror interference, which simplifies the configuration of the whole exposure system and guarantees the overall area of the fabricated 2D crossed grating. The polarization modulation effects at arbitrary incident angles were precisely quantified to determine the fabricability of high-uniformity 2D crossed gratings with different grating periods. It should be noted that this polarized holographic lithography system for period-tunable 2D microstructure patterning could be extended for application to nanophotonics, e.g., photonic crystals and photodetectors where micro- and nanopillar array structures are required.

## Conclusion

We conducted theoretical and experimental investigations of the feasibility of using an orthogonal two-axis Lloyd’s mirror interferometer to fabricate high-uniformity 2D crossed-grating patterns with different grating periods based on the polarization modulation approach. The interferometer unit, consisting of a grating holder and two mirrors with a corner-cube-like configuration, can divide a single beam into three sub-beams as per the wavefront-division scheme to realize 2D crossed interference fringes. We analyzed the nonorthogonality of the polarization vectors to precisely determine the interference level between any two sub-beams. A polarization modulation model was established considering two conditions: (a) eliminating unexpected interference between the two reflected sub-beams and (b) providing identical interference intensities between the incident sub-beam and the two reflected sub-beams. Two routes were adopted to obtain the optimal combination of the initial-polarization angles based on the order in which the two conditions were implemented. We found that route 1 ((a)→(b)) more efficiently eliminates the influence of additional interference in the incident angle range of [0°–43.8°], whereas route 2 ((b)→(a)) affords a performance similar to route 1 in the remaining incident angle range of [43.8°–90°]. Furthermore, there is a stable incident angle regime in the range of [48°–90°] that guarantees the fabricability of high-uniformity 2D interference fringes. For the incident angle range considered, the fabricable periods of the 2D crossed grating obtained with our 442-nm laser are >467.2 nm. This result implies that the conventional requirements of optical encoder systems with a grating period range from 500 to 1500 nm can be easily achieved with our system. At each incident angle, two optimal initial-polarization-angle combinations of the three sub-beams exist in the negative and positive polarization angle ranges of [−90°–0°] and [0°–90°].

In our experiments, we adopted route 2 as the actual implementation scheme because it utilizes a simpler optical system and provides identical interference intensity contrasts. We established an exposure setup with the orthogonal interference unit and two additional HWPs to adjust the initial-polarization angles of the sub-beams. For different initial-polarization-state combinations of sub-beams 1–3, we obtained fabricated 2D crossed gratings with different grating periods of 500, 750, 1000, 1250, and 1500 nm. Importantly, the experimental results closely match the simulated interference fringes. In conclusion, our systematic polarization manipulation method, as applied to the two-axis Lloyd’s mirror interference system, is a promising approach to fabricating high-uniformity 2D crossed gratings with a relatively large grating period range of 500 to >1500 nm. We have realized a rapid and stable approach for patterning period-tunable 2D-array microstructures with high uniformity based on the wavefront-division single-exposure scheme. Moreover, this polarized holographic lithography method is expected to be applicable to other multibeam interference lithography techniques.

It should be noted that the interference area of the three sub-beams in the orthogonal two-axis Lloyd’s mirror interferometer is limited by the light transmission areas of the HWPs in the optical system and the incident angles corresponding to the desired grating periods. In future studies, we plan to customize HWPs with larger light transmission areas and small frames at the edge to further enlarge the interference area of the three sub-beams for certain applications.

## Materials and methods

### Grating fabrication

In our experiment, we used silicon (Si) wafer with a surface roughness of 1 nm as a grating substrate to generate PR-based grating patterns. Prior to the PR spin-coating process, the Si substrate was dehydrated by means of a hot plate operated at 140° for 30 min to enhance the PR surface adhesion. The S1805-positive PR, diluted by propylene glycol methyl ether acetate at a volume ratio of 1:1, was spin-coated at 6000 rpm for 30 s to achieve a thickness of ~170 nm. This was followed by soft-baking the substrate with a hot plate at 100° for 3 min. To demonstrate the device fabrication capability with a large grating period range, the interference unit was rotated to incident angles of 51.3°, 65.4°, 71.8°, 75.5°, and 78°, which correspond to generated grating periods of 500, 750, 1000, 1250, and 1500 nm, respectively. After exposing the spin-coated PR to an incident dose of 27–36 mJ, the Si substrate with PR was immersed in 2.3% tetramethylammonium hydroxide solution for 18 s. After rinsing the Si substrate with deionized water for 10 s, hard-baking was performed with a hot plate at 110° for 10 min to solidify the PR grating patterns.
